# Knowledge, attitudes, and concerns about medical cannabis among U.S. healthcare professionals

**DOI:** 10.1186/s42238-026-00450-8

**Published:** 2026-05-29

**Authors:** Lakshmi Kumar, Erin Wang, David S. Mathai, C. Austin Zamarripa, Tory R. Spindle, Ryan Vandrey, Albert Garcia-Romeu

**Affiliations:** 1 Behavioral Pharmacology Research Unit, Department of Psychiatry and Behavioral Sciences, Johns Hopkins University School of Medicine, 5510 Nathan Shock Dr, Baltimore, MD 21224 USA; 2 Department of Psychiatry and Behavioral Sciences, Johns Hopkins University School of Medicine, Baltimore, MD USA; 3 Ethical Legal Implications of Psychedelics in Society Program, Center for Medical Ethics and Health Policy, Baylor College of Medicine, Houston, TX USA; 4 Psychiatry and Behavioral Sciences, Baylor College of Medicine, Houston, TX USA

**Keywords:** Cannabis, Cannabinoids, Marijuana, Medical cannabis, Medical marijuana

## Abstract

**Background:**

Cannabis is among the most widely consumed psychoactive substances, with increasing consumption due to growing legalization for medical and non-medical use. As access expands, healthcare providers are faced with clinical challenges, despite variability in knowledge and attitudes on cannabis use in clinical practice.

**Methods:**

We conducted an anonymous, cross-sectional web-based survey of U.S. healthcare professionals to assess knowledge and attitudes regarding cannabis use in clinical settings. Participants completed demographic items, self-report measures of cannabis-related beliefs and attitudes, and an objective knowledge assessment covering cannabis therapeutic indications, risks, and mechanisms of action. Descriptive analyses were conducted, followed by multivariable linear regression models examining whether demographic characteristics, knowledge, or concerns predicted openness to the clinical use of cannabis.

**Results:**

Among 879 respondents (71% female; 86% White; mean age = 46 years), 89% reported having patients who use cannabis. The sample included mental health professionals (29%), registered nurses (25%), physicians (18%), and advanced practice providers (15%). Participants rated their self-reported knowledge highest for cannabis’s risks (mean = 4.1/5), followed by therapeutic indications (mean = 4.0/5), and mechanisms of action (mean = 3.5/5). In contrast, objective knowledge check scores were generally low across these domains (13–64% correct). Personal experience (76%) and popular media (73%) were the most endorsed sources of cannabis-related knowledge. Overall, most respondents (87%) endorsed the therapeutic promise of cannabis, 74% reported openness to recommending medical cannabis, and 95% supported its legal medical use. Commonly cited concerns regarding clinical use of cannabis included lack of trained providers (35%), possible patient exploitation (22%), recreational misuse (21%), and risk of psychosis (20%). Greater openness to clinical use was associated with higher self-rated knowledge, younger age, professional role, and lower levels of concern.

**Conclusions:**

Although most respondents reported having patients who use cannabis and were largely supportive of medical cannabis use, objective knowledge gaps and limited formal training were evident. These findings suggest a need for structured clinical training on cannabis pharmacology, dosing, contraindications, and legal and ethical frameworks, as well as better monitoring of cannabis use to support safe and informed patient care.

**Supplementary Information:**

The online version contains supplementary material available at 10.1186/s42238-026-00450-8.

## Background

Cannabis is one of the most widely consumed psychoactive substances globally, with its consumption continuing to increase in part due to expanding legalization for both medical and non-medical (i.e., recreational) purposes (CDC 2024; Degenhardt et al. 2008; Pessar et al. 2025). In the United States (U.S.), most states have enacted laws permitting medical cannabis use for specific conditions, though there is significant variability in conditions approved, legal frameworks, and regulatory oversight (Mead 2019; Patton 2020). As medical cannabis legalization expands, healthcare providers are increasingly faced with patient inquiries and clinical decision-making challenges related to incorporating cannabis and cannabinoids into clinical practice, despite considerable variability in their knowledge, attitudes, and preparedness,

The growth of legal cannabis markets has led to a broad range of available products that differ in cannabinoid content, route of administration, formulation (e.g., oils, edibles, inhalants), and potency (MacCallum and Russo 2018; Wadsworth et al. 2022). Δ9-tetrahydrocannabinol (THC), the primary psychoactive constituent of cannabis, produces a wide range of effects, some of which are positive and/or therapeutic (e.g., euphoria, relaxed mood, analgesia, antiemesis, muscle relaxation) and some negative (e.g., anxiety, paranoia, nausea, cognitive impairment) (Cohen et al. 2019; Volkow et al. 2016). Cannabidiol (CBD), the second most prevalent bioactive compound in cannabis, does not produce the characteristic intoxicating effects of THC, but has shown some therapeutic potential (Bilbao and Spanagel 2022; Crippa 2018; Hoch et al. 2025; Solmi et al. 2023; White 2019), and is believed by many to modulate some of THC’s psychoactive effects (Freeman et al. 2019; Russo 2011). However, CBD is not without risk as recent studies have shown that oral CBD can significantly inhibit the metabolism of THC and many other medications (Bansal et al. 2023; Zamarripa et al. 2023) and chronic consumption of high doses of CBD requires monitoring for potential impacts on liver function (Eadie et al. 2024). In addition, potential therapeutic applications of other cannabinoids such as cannabinol (CBN), cannabigerol (CBG), Δ9-tetrahydrocannabivarin (THCV), and others have been explored and shown some promise (Bonn-Miller et al. 2024; Cuttler et al. 2024; Jadoon et al. 2016). These suggested therapeutic effects have contributed to growing interest in the inclusion of cannabis and cannabinoid products for medical purposes.

Medical cannabis refers to a broad category of cannabis- or cannabinoid-based products that are used with the intent to alleviate symptoms or manage health conditions, often without formal regulatory approval from agencies such as the U.S. Food and Drug Administration (FDA) (Ebbert et al. 2018). Due to cannabis’s classification as a Schedule I substance under U.S. federal law, it cannot currently be prescribed or dispensed through traditional pharmacies. Instead, patients must obtain certification from a registered healthcare professional, placing these providers in the unique position of facilitating patient access to a federally restricted substance while also navigating inconsistent state laws, limited training on cannabinoids, varying levels of scientific evidence, and complex ethical considerations surrounding patient care. Although 40 states presently permit the use of cannabis for conditions like chronic pain, nausea, and muscle spasticity, the supporting evidence for these uses remains mixed (Ebbert et al. 2018; Belendiuk et al. 2015; Bhaskar et al. 2021; NASEM 2017). Meanwhile, 24 states currently allow or regulate cannabis for non-medical use by adults, enabling individuals to access cannabis products without physician oversight and potentially use them for self-medication (Bhaskar et al. 2021; Wallis et al. 2022). Complicating this landscape further, the 2018 U.S. Agricultural Improvement Act (“Farm Bill”) removed hemp and its derivatives, defined as cannabis containing ≤ 0.3% Δ9-THC, from the list of controlled substances (US 2018). Up until recently, this bill created a legal loophole that lead to widespread availability of hemp-derived cannabinoid products even in states where cannabis remains illegal (Babalonis et al. 2021; Livingston et al. 2022). However, in November 2025, Congress enacted a full-year FY2026 Agriculture appropriations act (P.L. 119–37, Division B) that plans to re-instate federal controls on certain hemp-derived products, substantially revising the framework established by this 2018 Farm Bill and potentially closing this loophole. Together, these regulatory complexities and shifting legal frameworks place healthcare providers in a unique place regarding medical cannabis decision-making, highlighting the need to better understand their knowledge and perceptions in this evolving landscape.

Although several studies have examined healthcare professionals’ perceptions of medical cannabis (Carlini et al. 2017; Kruger et al. 2022; Melnikov et al. 2021; Philpot et al. 2019; Weisman and Rodríguez 2021; Worster et al. 2023), most have relied exclusively on self-reported attitudes and perceived knowledge. Few have included objective assessments of evidence-based understanding (e.g., (Rice et al. 2022), and none, to our knowledge, have compared subjective and objective measures of knowledge in the same sample. Prior work consistently shows that healthcare providers tend to have limited knowledge (both objective and self-reported) of the therapeutic applications and risks of medical cannabis, express openness to its clinical use, and report strong interest in additional education and training. The current study advances this literature by integrating objective knowledge checks (i.e., assessing participant’s accuracy regarding cannabis’s therapeutic indications, risks, and mechanisms of action), alongside subjective self-assessment ratings of perceived knowledge and attitudes. This allows for better characterization of actual versus perceived knowledge gaps among healthcare professionals.

Accordingly, the primary objective of the present study was to assess knowledge and attitudes regarding the inclusion of cannabis in medical and therapeutic settings among U.S. healthcare professionals. Ultimately, this research aims to present a more comprehensive picture of the current opportunities and challenges surrounding the integration of cannabis in medicine, with implications for clinical training, policymaking, and medical implementation.

## Methods

### Participants

Online recruitment advertisements were placed on the Johns Hopkins Center for Psychedelic and Consciousness Research website and on social media (i.e., Facebook, X, reddit, and Instagram), and institutionally approved emails were distributed to Johns Hopkins Medicine employees. Recruitment ads sought healthcare professionals (e.g., in a role such as physician, psychologist, counselor, social worker, therapist, nurse, physician’s assistant, or emergency medical technician, among others) willing to complete web-based questionnaires related to the medical use of cannabis and psychedelics (e.g., “Are you a healthcare professional? What are your opinions on medical use of cannabis?”). In the present study, eligible individuals were: (1) at least 18 years old, (2) could read and write English fluently, and (3) reported working in a clinical setting in the United States as a healthcare professional or mental health provider. Data were collected from December 15, 2021 to October 9, 2023. Of 2,212 total responses collected, 1,333 failed to meet established study inclusion criteria (details below), leaving a final analysis sample of 879 (40% of the original sample).

### Procedures

Interested participants were directed to a webpage with study inclusion and exclusion criteria and general information about study participation. Participants completed an anonymous online survey hosted on the secure web-based platform Qualtrics (Provo, UT). This study was considered exempt by the Johns Hopkins University School of Medicine Institutional Review Board (IRB) because it involved minimal risk as no personally identifiable information was collected. Participation was voluntary and participants received no compensation for survey completion. All research was performed in accordance with relevant guidelines/regulations and in accordance with the Declaration of Helsinki. The data presented here are part of a larger study characterizing knowledge and attitudes about various potential novel therapies including psilocybin, MDMA, ketamine, and cannabis. Results on psilocybin and MDMA were published separately (Wang et al. 2024).

Quality control measures were implemented throughout the survey. Survey answers were rejected if participants: (1) failed to pass an automated public Turing test (“CAPTCHA”) item, (2) answered any of the three attention check items incorrectly, (3) indicated that they completed the survey more than once assessed by self-report or if duplicate use of the same IP address was detected (yes/no), (4) indicated that they had issues completing the survey that would make their responses inaccurate or invalid (yes/no), (5) indicated that they had trouble understanding the questions in a way that would make their responses inaccurate or invalid (yes/no), (6) indicated that they did not answer honestly or to the best of their knowledge (yes/no), or (7) did not complete the entire survey. Only respondents that met all these criteria were included in the analyses presented here.

### Survey design

The survey was divided into several sections (see Supplement A for the cannabis-related survey instrument). The first section of the survey was composed of basic demographic questions (i.e., respondents’ age, gender, highest level of education, race, ethnicity, religious affiliation, household income, professional role, specialty [for physicians], year of training completed, geographic region of residence in the US, prescribing capabilities, current clinical practice, research involvement, or lifetime history of hallucinogen use). Professional roles were categorized as: Physician, Advanced Practice Provider (APP; i.e., Physician Assistant, Nurse Practitioner, Advanced Practice Registered Nurse), Registered Nurse (RN), Mental Health Professional (MHP; i.e., Psychologist, Counselor, Therapist, Social Worker), and Other (i.e., EMT, Pharmacist, Other).The next sections were structured in parallel, with each section containing questions about a particular substance: psilocybin, MDMA, ketamine, and cannabis. The cannabis section was divided into (1) an assessment of cannabis-related beliefs, attitudes, and experiences, and (2) an objective knowledge check.

In part (1), participants were asked to rate how strongly they agreed with statements representing their knowledge and attitudes about the clinical use and legal accessibility of cannabis on a 5-point Likert scale ranging from “strongly disagree” to “strongly agree.” Participants indicated, by selecting all options that applied from a checklist, where most of their knowledge on cannabis came from, which sources they would trust for information on the therapeutic use of cannabis, and in what settings it would be appropriate to administer cannabis clinically. The importance of potential concerns was collected on a 5-point Likert scale ranging from “not at all concerned” to “extremely concerned.” A free text box was provided for participants to share any additional concerns. Respondents were asked if they had ever seen someone under the influence of cannabis, with four possible responses (“no, never”; “yes, while they were seeking medical care”; “yes, in a recreational context”; or “yes, in a research/clinical setting”). Participants could select all responses that applied. If participants responded yes to the previous question, they were asked to describe the experience they observed on a 5-point scale ranging from “primarily positive” to “primarily negative.”

In part (2), the objective knowledge checks assessed respondents’ knowledge across three domains; (a) evidence-based therapeutic indications, (b) risks and adverse effects, and (c) primary mechanism of action of cannabis (see Supplemental materials for the full survey). For therapeutic indications, respondents were asked “Current data indicate cannabis and cannabinoids (e.g., THC, CBD) may be useful for treating (select all that apply)”, with correct responses being 1) muscle spasms, 2) pain symptoms and related disorders, and 3) nausea and vomiting. For risks and adverse effects, respondents were asked “Risks of cannabis may include (Select all that apply)”, with correct responses being 1) increased blood pressure and heart rate, 2) dizziness, 3) nausea, and 4) psychosis. For mechanism of action, respondents were asked “The primary mechanism by which cannabis is thought to work is (select all that apply)”, with the correct response being 1) activity at CB1 and CB2 receptors. A response was scored as correct only if participants selected all correct answers without choosing any incorrect ones. A final free text box was provided for participants to share any additional thoughts on the therapeutic use of cannabis. Before moving on to the next section, participants were provided peer reviewed information and resources on the substance queried in that section.

The last section of the survey was a 3-item quality check. Participants were asked if they had issues completing the survey, had trouble understanding the questions in a way that would make their responses inaccurate or invalid, or if they did not answer the questions honestly or to the best of their knowledge. Finally, three attention check questions were interspersed throughout the entire survey to further assess the validity of responses.

### Variables

Knowledge and attitude Likert scale responses were grouped into four categories: 1) self-rated knowledge, 2) openness to clinical use, 3) belief in therapeutic promise, and 4) support for legal access (see Supplement B for complete list). Internal consistency (Cronbach’s alpha) was calculated for Likert scale items measuring similar domains (knowledge, openness, belief in therapeutic potential, and legal access). Each of the three knowledge check items was scored dichotomously, with credit awarded only when all correct options were selected and no incorrect options were endorsed.

### Statistical analysis

Participant demographic characteristics were summarized using descriptive statistics (see Table 1). We performed descriptive analyses of healthcare providers’ attitudes, concerns, and knowledge about cannabis. Responses to item assessing personal and professional exposure, attitudes and perceived knowledge, concern ratings, objective knowledge performance, trusted sources of knowledge, and views of appropriate clinical administration settings were tabulated (see Table 2). Pearson correlation of self-rated knowledge and objective knowledge check scores were computed for cannabis. Knowledge and attitude ratings, concern ratings, and knowledge check scores by professional role were also calculated (see Table 3).

**Table 1:** Demographic characteristics

		***N*** ** = 879**
		Mean (SD)
Age (years)		45.5 (12.7)
		N (%)
Gender	Female	626 (71%)
	Male	236 (27%)
	Non-binary	15 (2%)
	Prefer not to answer	2 (< 1%)
Race	White	754 (86%)
	American Indian	3 (< 1%)
	Asian/Pacific Islander	27 (3%)
	Black	15 (2%)
	Multiracial	30 (3%)
	None of the above	32 (4%)
	Prefer not to answer	18 (2%)
Hispanic Ethnicity (yes/no)		67 (8%)
Region of Residence	Pacific	140 (16%)
	Rocky Mountains	58 (7%)
	Southwest	87 (10%)
	Northeast	196 (22%)
	Southeast	265 (30%)
	Midwest	121 (14%)
	Noncontiguous	12 (1%)
Highest Level of Education	Associate degree	52 (6%)
	Bachelor’s degree	172 (20%)
	Master's degree	336 (38%)
	Doctorate degree	235 (27%)
	Professional degree	71 (8%)
	Some college credit, no degree	6 (1%)
	Trade/technical/vocational training	7 (1%)
Profession	Physician	156 (18%)
	▪ Physician	156 (18%)
	Advanced Practice Provider	129 (15%)
	▪ Physician Assistant	23 (3%)
	▪ Nurse Practitioner	83 (9%)
	▪ Advanced Practice Registered Nurse	23 (3%)
	Registered Nurse	223 (25%)
	▪ Registered Nurse	223 (25%)
	Mental Health Professional	258 (29%)
	▪ Psychologist	55 (6%)
	▪ Counselor	51 (6%)
	▪ Therapist	86 (10%)
	▪ Social Worker	66 (8%)
	Other	113 (13%)
	▪ Emergency Medical Technician	12 (1%)
	▪ Pharmacist	16 (2%)
	▪ Other	85 (10%)
Prescribing Capability (yes/no)		291 (33%)
Currently Practicing Clinically (yes/no)		807 (92%)
Conducting Research (yes/no)		185 (21%)
Ever Taken a Hallucinogen (yes/no)		650 (73%)

**Table 2:** Knowledge on cannabis and appropriate settings for clinical administration

	***N*** ** = 879**
Self-Reported Knowledge^a^	Mean (SD)
▪ Therapeutic indications	4.0 (0.9)
▪ Risks and Adverse Effects	4.1 (0.8)
▪ Mechanism of Action	3.5 (1.0)
Perfect Responses on Objective Knowledge Checks^b^	Count (%)
▪ Therapeutic Indications	186 (21%)
▪ Risks and Adverse Effects	116 (13%)
▪ Mechanism of Action	558 (64%)
Current Knowledge Sources	Count (%)
▪ Academic literature	588 (67%)
▪ Popular media	637 (73%)
▪ Informal conversations	621 (71%)
▪ Past experience with patients	589 (67%)
▪ Colleagues	351 (40%)
▪ Formal clinical training	243 (28%)
▪ Conferences	350 (40%)
▪ Personal experience	668 (76%)
Trusted Knowledge Sources	Count (%)
▪ Academic research centers	826 (94%)
▪ Experienced clinicians	809 (92%)
▪ Professional organizations	705 (80%)
▪ Private training institutions	435 (50%)
▪ Pharmaceutical companies	240 (27%)
Appropriate Clinical Administration Settings	Count (%)
▪ Specialized clinic	765 (87%)
▪ Private practice	705 (80%)
▪ Patient's home (supervised)	657 (74%)
▪ Outpatient clinic	647 (74%)
▪ Home (unsupervised)	600 (68%)
▪ Inpatient setting	530 (60%)
▪ Detox facility	481 (55%)
▪ Emergency department	215 (25%)
▪ None	9 (1%)

^a^Rated on 5-point Likert scale ranging from 1 "strongly disagree" to 5 "strongly agree."

^b^Based on Objective Knowledge check items (see Supplement A)

**Table 3:** Knowledge, attitudes, and concerns regarding cannabis by profession

	**Mean (SD) ** ^**a**^
Self-rated Knowledge	**3.9 (0.8)**
Physicians	3.9 (0.8)
Advanced practice providers ^b^	3.9 (0.8)
Registered nurses	4.0 (0.8)
Mental health professionals ^b^	3.8 (0.8)
Other ^b^	4.1 (0.7)
Openness to Clinical Use	**4.1 (0.9)**
Physicians	3.9 (1.0)
Advanced practice providers	4.1 (0.9)
Registered nurses	4.4 (0.8)
Mental health professionals	3.9 (1.0)
Other	4.1 (0.9)
Belief in Therapeutic Promise	**4.4 (0.6)**
Physicians	4.3 (0.6)
Advanced practice providers	4.3 (0.6)
Registered nurses	4.6 (0.5)
Mental health professionals	4.4 (0.6)
Other	4.6 (0.6)
Support for Legal Access	**4.4 (0.7)**
Physicians	4.3 (0.8)
Advanced practice providers	4.2 (0.8)
Registered nurses	4.6 (0.6)
Mental health professionals	4.5 (0.6)
Other	4.6 (0.6)
Concerns	
*Lack of trained providers*	
Physicians	3.0 (1.2)
Advanced practice providers	3.0 (1.2)
Registered nurses	2.8 (1.2)
Mental health professionals	3.2 (1.1)
Other	2.8 (1.3)
*Financial cost/insurance coverage*	
Physicians	2.2 (1.1)
Advanced practice providers	2.6 (1.2)
Registered nurses	2.6 (1.2)
Mental health professionals	2.5 (1.2)
Other	2.4 (1.2)
*Administration to patients with contraindications*	
Physicians	2.3 (1.1)
Advanced practice providers	2.4 (1.1)
Registered nurses	2.3 (1.1)
Mental health professionals	2.7 (1.2)
Other	2.3 (1.3)
*Exploitation of patients*	
Physicians	2.5 (1.3)
Advanced practice providers	2.5 (1.3)
Registered nurses	2.3 (1.3)
Mental health professionals	2.6 (1.3)
Other	2.2 (1.3)
*Psychosis*	
Physicians	2.7 (1.3)
Advanced practice providers	2.5 (1.2)
Registered nurses	2.1 (1.1)
Mental health professionals	2.6 (1.2)
Other	2.2 (1.2)
*Time required to administer*	
Physicians	1.6 (1.0)
Advanced practice providers	1.7 (0.9)
Registered nurses	1.4 (0.8)
Mental health professionals	1.7 (1.0)
Other	1.6 (0.9)
*Stigma*	
Physicians	1.9 (1.1)
Advanced practice providers	2.0 (1.1)
Registered nurses	1.8 (1.1)
Mental health professionals	2.0 (1.0)
Other	2.0 (1.1)
*Recreational use/misuse*	
Physicians	2.7 (1.2)
Advanced practice providers	2.7 (1.2)
Registered nurses	2.1 (1.1)
Mental health professionals	2.6 (1.2)
Other	2.2 (1.2)
*Addiction*	
Physicians	2.6 (1.1)
Advanced practice providers	2.6 (1.2)
Registered nurses	2.1 (1.0)
Mental health professionals	2.6 (1.1)
Other	2.2 (1.1)

^a^Rated on 5-point Likert scale ranging from 1 "strongly disagree" to 5 "strongly agree."

^b^Professions were grouped as such: Advanced Practice Provider = Physician Assistant, Nurse Practitioner, Advanced Practice Registered Nurse. Mental Health Professional = Psychologist, Counselor, Therapist, Social Worker. Other = all others (e.g. EMT, pharmacist)

Finally, multivariable linear regression was performed to conduct a predictive analysis of healthcare providers’ openness to clinical use of cannabis based on their demographic characteristics, personal experience, self-rated knowledge, concern ratings, and profession (see Table 4). Covariates included age, profession, personal experience using cannabis, self-rated cannabis knowledge, and average self-rated cannabis concern. Supplementary analyses were additionally conducted including sex, race, and level of education as additional covariates (see Table S2). Additional analyses were conducted using the same modeling approach to evaluate predictors of objective cannabis knowledge using the same set of demographic and experiential variables (see Table 5 and S3).

**Table 4:** Multivariable linear regression model: openness to cannabis use in treatment

Coefficient	Estimate	Standard Error	t value	Pr(>|t|)	Significance
Intercept	2.94	0.23	12.68	<.001	***
Profession: APP	0.24	0.11	2.29	.022	*
Profession: RN	0.42	0.09	4.55	<.001	***
Profession: MHP	0.03	0.09	0.32	.746	
Profession: Other	0.11	0.11	0.99	.323	
Age Group: 30–39	−0.19	0.11	−1.83	.067	
Age Group: 40–49	−0.23	0.11	−2.14	.033	*
Age Group: 50–59	−0.32	0.11	−2.79	.005	**
Age Group: 60–69	−0.31	0.13	−2.41	.016	*
Age Group: 70 +	−0.31	0.19	−1.63	.103	
Cannabis experience: PNA	−0.22	0.21	−1.01	.315	
Cannabis experience: Yes	0.03	0.070	0.486	.627	
Self-rated cannabis knowledge	0.35	0.04	9.04	<.001	***
Mean cannabis concern score	−0.09	0.04	−2.11	.035	*

Reference groups: Profession = Physicians; Age = 18–29; Cannabis experience = No

*APP* Advance practice provider, *RN* Registered nurse, *MHP* Mental health professional, *PNA* Prefer not to answer

Significance codes: * = 0.05, ** = 0.01, *** = 0.001

**Table 5:** Multivariable linear regression model: cannabis objective knowledge

Coefficient	Estimate	Standard Error	t value	Pr(>|t|)	Significance
Intercept	0.87	0.19	4.54	<.001	***
Profession: APP	− 0.31	0.09	− 3.64	<.001	***
Profession: RN	− 0.60	0.08	− 7.90	<.001	***
Profession: MHP	− 0.72	0.07	− 9.90	<.001	***
Profession: Other	− 0.58	0.09	− 6.43	<.001	***
Age Group: 30–39	0.05	0.09	0.51	.611	
Age Group: 40–49	− 0.10	0.09	− 1.07	.285	
Age Group: 50–59	− 0.16	0.10	− 1.63	.103	
Age Group: 60–69	− 0.07	0.11	− 0.67	.503	
Age Group: 70 +	− 0.24	0.15	− 1.61	.107	
Cannabis experience: Yes	0.18	0.06	3.24	.001	**
Cannabis experience: PNA	0.12	0.17	0.71	.481	
Self-rated cannabis knowledge	0.07	0.03	2.15	.032	*
Mean cannabis concern score	0.11	0.03	3.25	.001	**

*APP* Advance practice provider, *RN* Registered nurse, *MHP* Mental health professional, *PNA* Prefer not to answer

Reference groups: Profession = Physicians; Age = 18–29; Cannabis experience = No

Significance codes: * = 0.05, ** = 0.01, *** = 0.001

## Results

### Participants

Of 879 participants, 626 (71%) were female, 754 (86%) were White, and 30% resided in the Southeast (Table 1). Mean (SD) age was 46 (Solmi et al. 2023) years. MHPs comprised the largest professional group (258, 29%), followed by RNs (*n* = 223, 25%) and physicians (*n* = 156, 18%). The most represented physician specialties were psychiatry (*n* = 42, 27%), family medicine (*n* = 26, 17%), and internal medicine (*n* = 25, 16%). A total of 291 participants (33%) held prescribing capabilities, 807 (92%) currently practiced clinically, and 185 (21%) conducted research (not mutually exclusive).

A total of 778 participants (89%) reported that they have patients that currently use cannabis. Most participants reported having seen someone under the influence of cannabis (*n* = 848, 97%) in a recreational context. Observed experiences were predominantly positive (*n* = 640, 73%), but negative experiences were also reported (*n* = 79, 9%).

### Self-reported and objective knowledge

Participants rated how strongly they agreed with statements representing their knowledge and attitudes about the clinical use and legal accessibility of cannabis on a 5-point Likert scale ranging from 1 (strongly disagree) to 5 (strongly agree). On average, participants rated their self-reported knowledge as highest regarding cannabis’s risks and adverse effects (mean = 4.1), followed by therapeutic indications (mean = 4.0), and finally on mechanism of action (mean = 3.5) (Table 2).

Objective knowledge check scores did not align with self-ratings. When examined by domain, only 116 (13%) of respondents correctly answered the knowledge check of risks and adverse effects of cannabis, despite self-rated knowledge being the highest in this domain. Additionally, 186 (21%) respondents correctly answered the knowledge check on therapeutic indications, and 558 (64%) on mechanisms of action. Only 22 (3%) respondents answered all 3 knowledge check questions correctly, while 241 (27%) respondents answered all 3 questions incorrectly. When the criteria were broadened to include participants who selected all correct responses but also included one or more incorrect options, the number of respondents who met criteria increased to 126 (14%) for knowledge of risks and adverse effects, 626 (71%) for therapeutic indications, and 615 (70%) for mechanism of action. Under these inclusive criteria, 85 (10%) respondents answered all 3 knowledge check questions correctly, while only 65 (7%) respondents answered all three incorrectly. Self-rated and objective knowledge scores were weakly but significantly correlated (*r* = 0.08; *p* < 0.05).

### Primary and trusted sources of knowledge

Respondents most frequently cited personal experience (76%), popular media (73%), and informal conversations (71%) as current actual sources of knowledge, followed by past patient experience (67%) and academic literature (67%) (Table 2).

By contrast, the most trusted sources of knowledge for training and information on therapeutic cannabis use were reported being academic research centers (94%), experienced clinicians or practitioners (92%), and professional organizations (80%). Only 27% of respondents stated they would trust pharmaceutical companies to provide reliable information on cannabis.

### Attitudes regarding cannabis

Overall internal consistency was high within grouped knowledge and attitude domains, with Cronbach’s α ranging from 0.80 to 0.83 (Supplemental Table S1).

#### Therapeutic promise

Belief in therapeutic promise of cannabis was high across all professions (Table 3). Overall, mean (SD) ratings for belief in therapeutic promise was 4.4 (0.6), indicating an average response between “Agree” and “Strongly Agree.” Specifically, 87% believed cannabis can be delivered safely in clinical settings and 87% endorsed its therapeutic promise. Finally, 96% supported further cannabis research.

#### Openness to clinical use

Openness to clinical use was rated a mean (SD) of 4.1 (0.9) for cannabis. A majority endorsed openness to recommending medical cannabis (74%) and a majority also expressed an interest in further training to use cannabis (78%) in their practice. Openness to clinical use of cannabis was lower among physicians (mean = 3.9) and mental health professionals (mean = 3.9) compared to other professions on average (mean = 4.1–4.4).

#### Support for legal access

Most participants (95%) supported legal medical use of cannabis, and slightly fewer (86%) felt cannabis should be legally accessible for recreational/non-medical use. Finally, 88% noted support for legal access to cannabis for religious use. Mean support for legal access to cannabis (i.e., across all settings) was higher for other professionals, RNs, and MHPs than for physicians and APPs.

#### Concerns about clinical use of cannabis

**Fig. 1:**
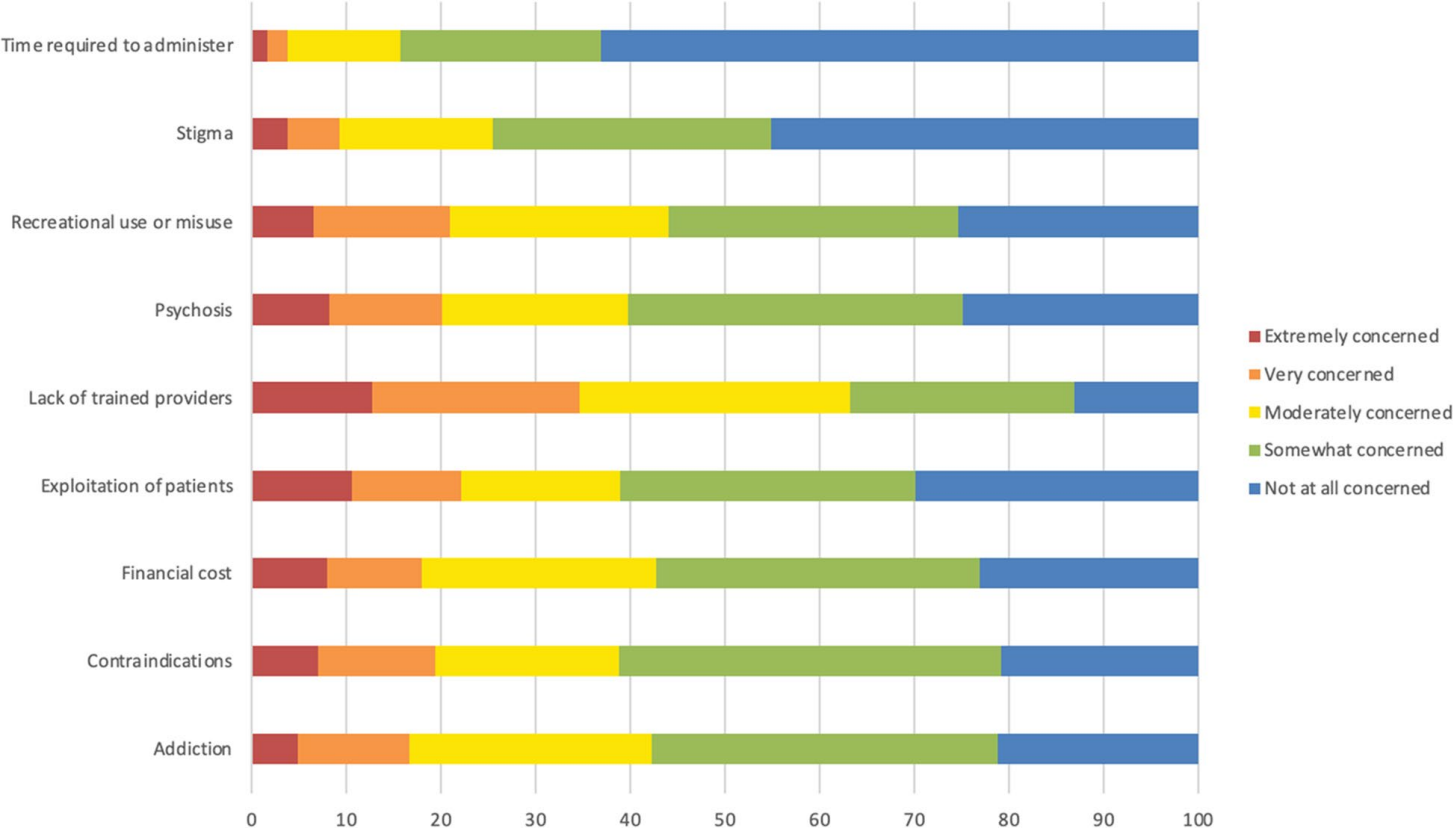
Concern ratings for cannabis. This figure shows the proportion of respondents stating their level of concern (1 = Not at all concerned and 5 = Extremely concerned) for each of the following potential issues related to cannabis-assisted therapy.

The most highly endorsed concerns regarding clinical use of cannabis were lack of trained providers (35% responded “extremely concerned” or “very concerned”), followed by potential exploitation of patients (22%), recreational use and misuse of cannabis (21%), potential for inducing psychosis (20%), potential harms to patients with contraindications (19%), financial costs/insurance coverage of cannabis treatment (18%), and the addictive potential of cannabis (17%). Stigma surrounding cannabis (9%) and amount of time needed for cannabis treatment (4%) were rated as less concerning (see Fig. 1).

MHPs reported the highest concern scores on average, followed by APPs and physicians, then RNs (Table 3). MHPs had greater concerns about lack of trained providers, administration to patients with contraindications, exploitation of patients, potential for inducing psychosis, recreational use and misuse of cannabis, and addiction.

### Appropriate settings for cannabis therapies

Respondents regarded specialized clinics (87%) to be the most appropriate clinical setting for the administration of cannabis, followed by private practice (80%), patient’s home with supervision (74%), outpatient clinics (74%), and patient’s home with without supervision (68%).

### Predictive models

Multivariable linear regression models were used to determine how professional role, demographic characteristics, knowledge, and personal experience predict healthcare professionals’ openness to the clinical use of cannabis. Demographic variables predicted a modest proportion of total variance (R2 = 0.159; Table 4. Greater openness to clinical use of cannabis was significantly associated with higher self-rated cannabis knowledge. Openness significantly decreased as age increased, with respondents aged 18–29 years reporting the greatest openness. APPs and RNs were more open to the clinical use of cannabis compared to physicians. Greater concern about cannabis was significantly associated with lower openness to its clinical use. Separate multivariable regression models examined predictors of healthcare professionals’ objective cannabis knowledge (Table 5). Demographic characteristics predicted a small proportion of total variance (R2 = 0.141). Higher objective cannabis knowledge was significantly associated with greater self-rated cannabis knowledge, personal experience using cannabis, and greater concern about cannabis. APPs, RNs, MHP, and other health professionals demonstrated less objective cannabis knowledge than physicians. Age was not a significant predictor of objective cannabis knowledge.

## Discussion

The present study aimed to characterize knowledge and attitudes on medical cannabis among a convenience sample of U.S. healthcare providers, broadly defined. It is important to note that this was a self-selected demographically homogenous sample. Our intentions for gathering these data were primarily to inform ongoing efforts in policymaking, institutional decision-making, and clinical training and practice surrounding medical cannabis. Key findings indicated that most respondents (> 85%), regardless of their role in healthcare, believed cannabis to have legitimate therapeutic uses and had patients who used cannabis. Furthermore, most participants were supportive of legal access to medical cannabis, open to using it in their own practice, and interested in receiving further training to do so. Consistent with these descriptive findings, multivariable regression models showed that openness to clinical use of cannabis was positively associated with greater self-related cannabis knowledge and negatively associated with greater concern about cannabis, suggesting that both perceived knowledge and perceived risk can shape clinical attitudes. Notably, even among highly trained medical professionals (e.g., nurses, physicians, pharmacists), less than a third of participants endorsed formal clinical training as a primary source of knowledge for whether or how to integrate medical cannabis in their practice. Instead, many reported relying on personal experience, popular media, and informal conversations as the primary sources of information on the topic, highlighting a critical gap between clinical practice and institutional training.

While participants’ self-reported knowledge did correlate significantly, though weakly, with their performance on a brief objective knowledge check assessing therapeutic indications, risks, and mechanisms related to medical cannabis, there were inconsistencies in self-report vs. objective knowledge. Specifically, although respondents rated themselves as most knowledgeable about cannabis’s risks, objective knowledge scores were lowest in this domain, suggesting overconfidence and a disconnect between perceived and actual awareness about cannabis-related harms. This discrepancy is further contextualized by regression findings indicating that greater objective cannabis knowledge was associated not only with higher self-rated knowledge, but also greater concern about cannabis, suggesting that increased factual understanding may heighten awareness rather than necessarily openness to clinical use. This misalignment is especially concerning given clinicians’ roles in advising patients on safe and effective cannabis use. Similarly, although self-rated knowledge of therapeutic indications for medical cannabis was also high (rated 4 on a 5-point scale), less than a quarter of respondents correctly identified evidence-based indications on objective knowledge checks. These findings highlight the need for more effective educational interventions that go beyond passive exposure to medical cannabis related information and actively test, reinforce, and update clinical knowledge. Overall, physicians and pharmacists tended to provide more correct responses to objective knowledge checks, consistent with their professional roles in prescribing and/or administering medications. Indeed, regression models showed that physicians performed better than all other health professionals on objective cannabis knowledge questions. Academic research centers, experienced clinicians, and professional organizations were cited as the most trusted sources of information on medical cannabis, suggesting an important opportunity for these groups to develop and offer structured trainings on medical cannabis. Although some healthcare training programs have begun to incorporate cannabis-related content, there is a general lack of structured and standardized cannabis education in health curricula. Additionally, healthcare trainees report limited formal instruction and low confidence in counseling patients about medical cannabis (Zolotov et al. 2021).

A perceived lack of adequately trained providers, potential patient exploitation, and cannabis misuse among patients were commonly noted concerns among healthcare providers. These concerns likely reflect perceived gaps in clinical preparedness and confidence regarding medical cannabis use and are particularly relevant when considering the growing complexity in clinical cannabis care, including a growing variety of available cannabinoid products (Wadsworth et al. 2022), the presence of unregulated cannabis products with documented heavy metal contamination and other toxic impurities (Dryburgh et al. 2018; Gardener et al. 2022), rising cannabis use (Mattingly et al. 2024), and increasing rates of cannabis use disorder (CUD) (Haley 2024). These findings highlight the clear need for structured, evidence-based cannabis education across all levels of healthcare (Marcu 2025). Clinician training should include legal and regulatory frameworks, therapeutic dosing, pharmacokinetics, drug interactions, management of adverse effects, and effective patient communication (Kruger et al. 2022; Baral et al. 2025; Cronin et al. 2025).

Respondents with higher self-rated knowledge and younger age were more likely to express openness to clinical use of cannabis in their practice, possibly reflecting generational differences in medical training as well as general attitudes. Unsurprisingly, those who reported greater concerns were less open to recommending medical cannabis in their practice, with physicians endorsing less openness generally than APPs and registered nurses. Across the board, nearly all participants (96%) supported additional research on cannabis, highlighting a large need for rigorous sources of clinical data regarding both risks and therapeutic applications. Indeed, 76% of respondents reported that their knowledge of cannabis primarily came from personal experience rather than formal education or clinical training. Given the extensive transition to electronic health records (EHRs), we recommend providers be diligent in asking patients about cannabis use and making detailed documentation in the EHR of their cannabis use status, including the type of cannabis (THC-dominant, CBD-dominant, other), source (dispensary vs. unregulated source), route of administration, dose, and frequency of use.

These results are limited by several factors. First, the study used a convenience sample of individuals who responded to online advertising and social media posts related to psychedelic medicines and most participants indicated openness to using cannabis or other cannabinoids therapeutically in their clinical practice. Thus, data likely reflect interest and potentially favorable bias among the sample towards novel therapies or plant-based medicines such as cannabis. Because this survey relied on unverified self-report data, results cannot be strongly substantiated or support robust conclusions. However, survey data showed good internal consistency as well as conceptually compatible associations (e.g., lower openness to clinical use among respondents who reported greater concerns about medical cannabis), providing some evidence for valid responses over and above other data integrity measures. Second, given that eligibility was determined solely by self-report, we were unable to verify that participants were in fact healthcare professionals, which limits the confidence with which findings can be attributed to a true healthcare provider sample. Third, the entire questionnaire was developed specifically for this study, meaning that the self-rated and objective knowledge questions have not undergone any formal validation. Although our findings revealed inconsistencies between self-reported and objective cannabis knowledge, future research would benefit from the development and psychometric validation of standardized tools to assess self-perceived and objective cannabis-related knowledge and attitudes across healthcare professionals. Fourth, the categorization of participants’ professional roles presents a limitation. Participants selected their role from a predefined list of broad designations (e.g., therapist, counselor), which include a wide range of training backgrounds, licensing requirements, and clinical responsibilities. Furthermore, these roles were classified as general categories (e.g., Mental Health Professionals) for analyses. Thus, our findings cannot be used to draw meaningful conclusions across professions. Future research should collect more specific information about professional credentials and responsibilities. Fifth, because the sample was primarily comprised of white female respondents, the data may not generalize broadly to U.S. healthcare providers. Additionally, the heterogeneity of the sample, which included a wide range of healthcare providers, cannot be taken to represent the perceptions of any specific healthcare profession. Nevertheless, the survey was designed to gather information from a broad base of professionals working in the healthcare space in part to examine potential differences across sectors.

## Conclusions

As cannabis use grows in clinical settings (Rhee and Rosenheck 2023), understanding healthcare providers’ knowledge, attitudes, and preparedness to guide patients regarding cannabis consumption is critical. While some specialties, particularly psychiatry and behavioral health, suggest providers may hold generally favorable attitudes toward cannabis therapies (Kruger et al. 2022; Philpot et al. 2019; Russo et al. 2024), clinical training and familiarity with the evidence base on medical cannabis use are often inconsistent. This gap presents both opportunities and challenges: opportunities for improved clinical education and training programs, for improved patient care, and to systematically collect data related to cannabis consumption behavior to establish clinical practice guidelines, and challenges arising from widespread use among patients who currently rely on healthcare providers with limited formal education on cannabis, or who are using cannabis without the knowledge of their providers. Given the expanding legal access to medical cannabis and growing patient interest, next steps should move beyond documenting knowledge gaps towards developing and evaluating standardized, evidence based educational curricula in medical, nursing, and pharmacy training programs. These curricula should integrate cannabis pharmacology, dosing principles, contraindications, and legal/ethical frameworks. Additionally, collaboration between healthcare institutions, regulatory agencies, and research centers would be helpful to track cannabis use, adverse events, and treatment outcomes across populations. These types of data will be essential for developing evidence-based prescribing and harm-reduction guidelines as cannabis product diversity and potency continue to expand.

## Electronic Supplementary Material

Below is the link to the electronic supplementary material.

Supplementary Materials. Includes Supplement A, Supplement B, Table S1, Table S2, and Table S3.

Supplement A. Full survey instrument

## Data Availability

The data used and/or analyzed during the current study are available from the corresponding author on reasonable request.
