# Supplementary material for: Knowledge, attitudes, and concerns about medical cannabis among U.S. healthcare professionals
Source: J Cannabis Res. 2026 May 29;8:88. doi: 10.1186/s42238-026-00450-8 (PMC13425986; doi:10.1186/s42238-026-00450-8)
Supplement: Supplementary file 1 — Supplementary Materials. Includes Supplement A, Supplement B, Table S1, Table S2, and Table S3. [file 42238_2026_450_MOESM1_ESM.docx]

**Supplementary Materials**

**Supplement A: Survey Instrument (uploaded as separate document)**

**Supplement B: Grouped Knowledge and Attitude Likert Scale Questions**

Self-rated knowledge:

1. I have good knowledge of the potential therapeutic uses of cannabis.
2. I have good knowledge of the risks and side effects of cannabis.
3. I have good knowledge of the pharmacology of cannabis.

Openness to clinical use:

1. I would be open to using cannabis clinically.
2. I would like to receive training on using cannabis as a treatment with my patients.

Belief in therapeutic promise:

1. Cannabis can be delivered safely in a clinical setting.
2. Cannabis shows promise in treating psychiatric disorders.
3. Cannabis deserves further research for treatment of psychiatric disorders.

Support for legal access:

1. Cannabis should be legally accessible for spiritual/religious use.
2. Cannabis should be legally accessible for recreational/non-medical use.
3. Cannabis should be legally accessible for supervised medical use.

**Table S1. Internal Consistency Across Domains (Cronbach's α)**

| **Domains** | **Cannabis** |
| --- | --- |
| Self-rated knowledge | 0.83 |
| Openness to clinical use | 0.81 |
| Belief in therapeutic promise | 0.80 |
| Support for legal access | 0.81 |

**Table S2. Multivariable linear regression model: Openness to cannabis use in treatment.**

| **Coefficient** | **Estimate** | **Standard Error** | **t value** | **Pr(>\|t\|)** | **Significance** |
| --- | --- | --- | --- | --- | --- |
| Intercept | 2.85 | 0.29 | 9.98 | <.001 | *** |
| Profession: APP | 0.27 | 0.13 | 2.11 | .035 | * |
| Profession: RN | 0.56 | 0.14 | 3.91 | <.001 | *** |
| Profession: MHP | 0.04 | 0.11 | 0.36 | .722 |  |
| Profession: Other | 0.18 | 0.13 | 1.39 | .165 |  |
| Age: 30-39 | -0.20 | 0.11 | -1.88 | .060 |  |
| Age: 40-49 | -0.23 | 0.11 | -2.14 | .033 | * |
| Age: 50-59 | -0.34 | 0.12 | -2.96 | .003 | ** |
| Age: 60-69 | -0.32 | 0.13 | -2.45 | .013 | * |
| Age: 70+ | -0.36 | 0.19 | -1.84 | .066 |  |
| Sex: Male | 0.11 | 0.07 | 1.46 | .144 |  |
| Sex: Non-binary | 0.24 | 0.24 | 1.00 | .316 |  |
| Sex: Transgender | 1.49 | 0.89 | 1.69 | .092 |  |
| Sex: PNA | -0.60 | 0.66 | -0.92 | .359 |  |
| Race: Black | -0.35 | 0.23 | -1.50 | .133 |  |
| Race: Indian | -0.25 | 0.26 | -0.96 | .337 |  |
| Race: American Indian | -1.09 | 0.51 | -2.16 | .031 | * |
| Race: Asian Pacific Islander | 0.11 | 0.23 | 0.48 | .633 |  |
| Race: Multiracial | -0.07 | 0.16 | -0.46 | .649 |  |
| Race: None apply | 0.13 | 0.16 | 0.79 | .429 |  |
| Race: PNA | 0.24 | 0.22 | 1.07 | .285 |  |
| Education: No degree* | -0.03 | 0.39 | -0.08 | .940 |  |
| Education: Trade/Technical | -0.17 | 0.36 | -0.48 | .635 |  |
| Education: Bachelor’s degree | -0.12 | 0.14 | -0.86 | .390 |  |
| Education: Master’s degree | 0.07 | 0.15 | 0.45 | .651 |  |
| Education: Professional degree | 0.15 | 0.19 | 0.79 | .429 |  |
| Education: Doctoral degree | 0.00 | 0.17 | 0.02 | .986 |  |
| Cannabis experience: PNA | -0.27 | 0.21 | -1.25 | .213 |  |
| Cannabis experience: Yes | 0.02 | 0.07 | 0.35 | .730 |  |
| Self-rated cannabis knowledge | 0.36 | 0.04 | 8.91 | <.001 | *** |
| Mean cannabis concern score | -0.09 | 0.04 | -2.05 | .041 | * |

Note. APP = Advance practice provider; RN = Registered nurse; MHP = Mental health professional; PNA = Prefer not to answer.

Reference groups: Profession = Physicians; Age = 18-29; Sex = Female; Race = Caucasian; Education = Associate’s degree; Cannabis experience = No.

Significance codes: * = 0.05, ** = 0.01, *** = 0.001

^a^Some college credit

Residual standard error: 0.869 on 848 degrees of freedom

Multiple R-squared: 0.180, Adjusted R-squared: 0.151

F-statistic: 6.22 on 30 and 848 DF, p-value: < 2.2e-16

**Table S3. Multivariable linear regression model: Cannabis Objective Knowledge.**

| **Coefficient** | **Estimate** | **Standard Error** | **t value** | **Pr(>\|t\|)** | **Significance** |
| --- | --- | --- | --- | --- | --- |
| Intercept | 0.52 | 0.23 | 2.23 | 0.026 | * |
| Profession: APP | -0.18 | 0.10 | -1.81 | 0.071 |  |
| Profession: RN | -0.60 | 0.09 | -6.69 | 0.000 | *** |
| Profession: MHP | -0.38 | 0.10 | -3.67 | 0.000 | *** |
| Profession: Other | -0.38 | 0.12 | -3.28 | 0.001 | ** |
| Age: 30-39 | -0.02 | 0.09 | -0.24 | 0.809 |  |
| Age: 40-49 | -0.15 | 0.09 | -1.56 | 0.118 |  |
| Age: 50-59 | -0.21 | 0.10 | -2.12 | 0.034 | * |
| Age: 60-69 | -0.17 | 0.11 | -1.58 | 0.115 |  |
| Age: 70+ | -0.35 | 0.15 | -2.37 | 0.018 | * |
| Sex: Male | 0.20 | 0.06 | 3.46 | 0.001 | *** |
| Sex: Non-binary | -0.11 | 0.19 | -0.57 | 0.572 |  |
| Sex: Transgender | 1.24 | 0.71 | 1.74 | 0.082 |  |
| Sex: PNA | -0.40 | 0.53 | -0.77 | 0.442 |  |
| Race: Black | 0.04 | 0.19 | 0.23 | 0.816 |  |
| Race: Indian | -0.41 | 0.21 | -1.97 | 0.050 | * |
| Race: American Indian | 0.48 | 0.41 | 1.18 | 0.240 |  |
| Race: Asian Pacific Islander | -0.03 | 0.19 | -0.18 | 0.860 |  |
| Race: Multiracial | -0.06 | 0.13 | -0.43 | 0.669 |  |
| Race: None apply | -0.04 | 0.13 | -0.30 | 0.766 |  |
| Race: PNA | -0.23 | 0.18 | -1.28 | 0.203 |  |
| Education: No degree^a^ | -0.78 | 0.31 | -2.52 | 0.012 | * |
| Education: Trade/Technical | -0.55 | 0.29 | -1.91 | 0.057 |  |
| Education: Bachelor’s degree | 0.13 | 0.11 | 1.16 | 0.247 |  |
| Education: Master’s degree | 0.20 | 0.12 | 1.60 | 0.109 |  |
| Education: Professional degree | 0.28 | 0.15 | 1.84 | 0.067 |  |
| Education: Doctoral degree | 0.30 | 0.13 | 2.22 | 0.027 | * |
| Cannabis experience: PNA | 0.10 | 0.17 | 0.55 | 0.581 |  |
| Cannabis experience: Yes | 0.18 | 0.06 | 3.21 | 0.001 | ** |
| Self-rated cannabis knowledge | 0.08 | 0.03 | 2.58 | 0.010 | ** |
| Mean cannabis concern score | 0.10 | 0.03 | 3.11 | 0.002 | ** |

Note. APP = Advance practice provider; RN = Registered nurse; MHP = Mental health professional; PNA = Prefer not to answer.

Reference groups: Profession = Physicians; Age = 18-29; Sex = Female; Race = Caucasian; Education; Associate degree; Cannabis experience = No.

Significance codes: * = 0.05, ** = 0.01, *** = 0.001

^a^Some college credit

Residual standard error: 0.697 on 848 degrees of freedom

Multiple R-squared: 0.186, Adjusted R-squared: 0.158

F-statistic: 6.48 on 30 and 848 DF, p-value: < 2.2e-16
