# Supplementary material for: Knowledge, attitudes, and concerns about medical cannabis among U.S. healthcare professionals
Source: J Cannabis Res. 2026 May 29;8:88. doi: 10.1186/s42238-026-00450-8 (PMC13425986; doi:10.1186/s42238-026-00450-8)
Supplement: Supplementary file 2 — Supplement A. Full survey instrument [file 42238_2026_450_MOESM2_ESM.pdf]

Info/Eligibility

Thank you for your interest in this anonymous online survey study on healthcare professionals' knowledge and attitudes about hallucinogens. This research is being conducted by scientists at Johns Hopkins University School of Medicine and has been deemed "exempt" by the Johns Hopkins University Institutional Review Board (IRB), meaning it involves minimal risk and does not directly involve human subjects because no personally identifiable information (e.g., name, email address, IP address) will be collected in this survey. The greatest risks of this study include boredom or emotional discomfort when answering some questions.

Our Previous Research
Our research team has conducted surveys characterizing positive and challenging experiences after taking psychedelics and other drugs. We've also studied both potential clinical uses and risks of giving psilocybin to volunteers in our laboratory.

Purpose of This Study
The goal of this survey is to learn more about how much healthcare professionals (broadly defined) know about hallucinogens that are being used medically or considered for medical use. We also want to characterize how healthcare professionals view these substances and their potential risks and benefits. For the purposes of this survey, we will be asking specifically about psilocybin (found in "magic mushrooms"), 3,4-methylenedioxymethamphetamine (MDMA), ketamine, and cannabis. For the purposes of this survey, anyone who works in a clinical setting as a healthcare professional or mental health provider is welcome to respond. This includes, but is not limited to physicians, psychologists, counselors, social workers, therapists, nurses, physician's assistants, and emergency medical technicians. The present study is limited to individuals working in the United States.

Inclusion Criteria
You are invited to participate in this survey if you fulfill all of the three criteria listed below.
1) You are at least 18 years old.
2) You read and write English fluently.
3) You work in a clinical setting in the United States as a healthcare professional or mental health provider (i.e., in a role such as physician, psychologist, counselor, social worker,

therapist, nurse, physician's assistant, or emergency medical technician, among others).

What the Study Entails

Participation in this study involves filling out an online survey that will take approximately 20 minutes. You will be required to complete the survey in one sitting.

Why should I participate?

We believe that this study is scientifically important. We would like you to participate because we need to collect responses from many different people. You may find this survey interesting. Although there is no monetary compensation for participation, you will be making a unique and important contribution to science.

Is my participation confidential?

Yes. We do not collect identifying information such as your name, email address, or IP address. Your anonymous responses will be seen and analyzed by Johns Hopkins staff or representatives. The study data will be hosted on a secure website (www.Qualtrics.com). Qualtrics is a reputable and secure platform for conducting survey research online that is approved for use by Johns Hopkins. To further protect the confidentiality of participants, the results of most questions will be combined and presented as group data, rather than individual responses. We may quote from your textual responses. However, if you provide specific identifying information, we will edit your responses to protect your confidentiality.

Benefits

There is no direct benefit to you from being in this study. Responses from many different people and your input could advance our understanding of current knowledge and attitudes surrounding the medical use of hallucinogens. These opinions could be useful to inform future scientific research and policies regarding medical hallucinogen administration.

What kind of information will I be providing?

At the beginning of the survey, you will be asked questions about your background, demographics (age, race, etc.), and career training. We will also ask questions assessing your knowledge and attitudes regarding psilocybin, MDMA, ketamine, and cannabis and their potential risks and benefits in medical care. Finally, we will collect open-ended responses to gather other relevant comments or opinions regarding these substances and their use.

Risks/Discomforts

You may get tired or bored when you are completing the survey. You do not have to answer any question you do not want to answer.

What will become of the results from this study?

The researchers intend to publish the results from this study in the scientific literature and to present results at scientific meetings. We will also make the results publicly available by posting a notice of any scholarly publications on the website of the Johns Hopkins Center for Psychedelic and Consciousness Research (https://HopkinsPsychedelic.org).

Your responses to the survey will only be used if you complete the survey.

Your participation in this study is voluntary. Reviewing this information and completing the survey will serve as your consent to be in the study. Even after you begin the survey, you may stop answering the questions at any time. If you stop early, none of your responses will be used. At the end of the survey, you will have a final opportunity to consent, or not, to all of your responses being submitted. Some attention check items are included in the survey. If you respond incorrectly to any of these, none of your responses will be used.

How do I start?

You can begin the survey by clicking 'Begin survey' at the bottom of this page. This survey will take approximately 20 minutes. You will be required to complete the survey in one sitting. It is important that you complete the survey only once, and that you answer each question honestly and seriously. If you are not ready to complete the survey now, please return to this page at a time that is convenient for you.

You can exit the survey at any time by closing your browser window. If you exit the survey early, your responses will not be used.

During the survey, please do not hit the "back" button on your internet browser as it may erase your answers or prematurely terminate your session.

Contact Information

If you have any questions about this study, please feel free to contact the Principal Investigator Albert Garcia-Romeu, Ph.D. at (410) 550-1972 or agarci33@jhmi.edu.

The IRB can help you if you have questions about your rights as a research participant or if you have other questions, concerns or complaints about this research study. You may contact the IRB at 410-502-2092 or jhmeirb@jhmi.edu.

By clicking 'Begin survey' below, you affirm that:
• you have read the information above,
• you voluntarily agree to participate,
• you are at least 18 years old,
• you read and write English fluently,
• you work in a clinical setting in the United States as a healthcare professional or mental health provider (i.e., in a role such as physician, psychologist, counselor, social worker, therapist, nurse, physician's assistant, or emergency medical technician, among others).

Please click below to begin or exit the survey.

- ○ Begin survey
- ○ Exit survey

Have you completed this survey before?

- ○ Yes
- ○ No

Do you read, write, and speak English fluently?

- ○ Yes
- ○ No

Are you 18 years old or older?

- ○ Yes
- ○ No

Do you work in a clinical setting in the United States as a healthcare professional or mental health provider (i.e., in a role such as physician, psychologist, counselor, social

worker, therapist, nurse, physician's assistant, or emergency medical technician, among others)?

- ○ Yes
- ○ No

☐ I'm not a robot
reCAPTCHA
Privacy - Terms

For Individuals who do not meet inclusion criteria or opt to exit survey:

We sincerely appreciate your interest in this survey and your willingness to volunteer your time. Unfortunately, you do not currently meet all of the necessary criteria to participate.

In the future, we hope to initiate another version of this survey with less restrictive inclusion requirements. We hope that you may be interested in participating in such a survey in the future.

With gratitude for your interest,
The Johns Hopkins Center for Psychedelic and Consciousness Research

JOHNS HOPKINS
Center for Psychedelic & Consciousness Research

For Individuals who meet inclusion criteria and opt to begin survey:

Demographics

Please answer the following demographics questions.

Age

[dropdown]

Gender

- Male
- Female
- Transgender male
- Transgender female
- Non-binary
- ____________ Other
- Prefer not to answer

What is the highest degree or level of school you have completed?

- Some high school, no diploma
- High school graduate, diploma or the equivalent (GED)
- Some college credit, no degree
- Trade/technical/vocational training
- Associate degree
- Bachelor’s degree
- Master’s degree
- Professional degree
- Doctorate degree

Which of the following best describes your racial background? (select all that apply)

- White or European American
- Black or African American
- African ancestry
- American Indian or Alaska Native
- Asian Indian
- Chinese
- Filipino
- Japanese
- Korean
- Vietnamese
- Native Hawaiian Guamanian or Chamorro Samoan
- ____________ None of these apply to me. I describe my race/ethnicity as:

- [ ] Prefer not to answer

Are you Hispanic or Latino? ('Hispanic' or 'Latino' refers to a person of Cuban, Mexican, Puerto Rican, South or Central American, or other Spanish culture or origin, regardless of race.)

- ○ Yes
- ○ No
- ○ Prefer not to answer

How would you characterize your overall spiritual or religious belief system?

- ○ Agnostic (unsure)
- ○ Atheist (non-believer)
- ○ Buddhism
- ○ Christianity
- ○ Hinduism
- ○ Islam
- ○ Judaism
- ○ Sikhism
- ○ Spiritual but not religious
- ○ ________ Other (please specify)
- ○ Prefer not to answer

What is your total annual household income (in U.S. dollars)?

- ○ $0 to $9,999
- ○ $10,000 to $24,999
- ○ $25,000 to 49,999
- ○ $50,000 to 74,999
- ○ $75,000 to 99,999
- ○ $100,000 to 149,999
- ○ $150,000 and greater
- ○ Prefer not to answer

What is your current professional role?

- Physician
- Psychologist
- Counselor
- Social Worker
- Therapist
- Nurse Practitioner
- Registered Nurse
- Advanced Practice Registered Nurse
- Physician’s Assistant
- Emergency Medical Technician
- ____________ Other

Physician specialty

- Allergy and immunology
- Anesthesiology
- Cardiology
- Dermatology
- Diagnostic radiology
- Emergency medicine
- Family medicine
- Internal medicine
- Medical genetics
- Neurology
- Nuclear medicine
- Obstetrics and gynecology
- Ophthalmology
- Pathology
- Pediatrics
- Physical medicine and rehabilitation
- Preventive medicine
- Psychiatry
- Radiation oncology

- ◯ Surgery
- ◯ Urology
- ◯ [ ] Other
- ◯ N/A

In what year did you complete your professional training?

[ ]

In which U.S. region do you currently reside?

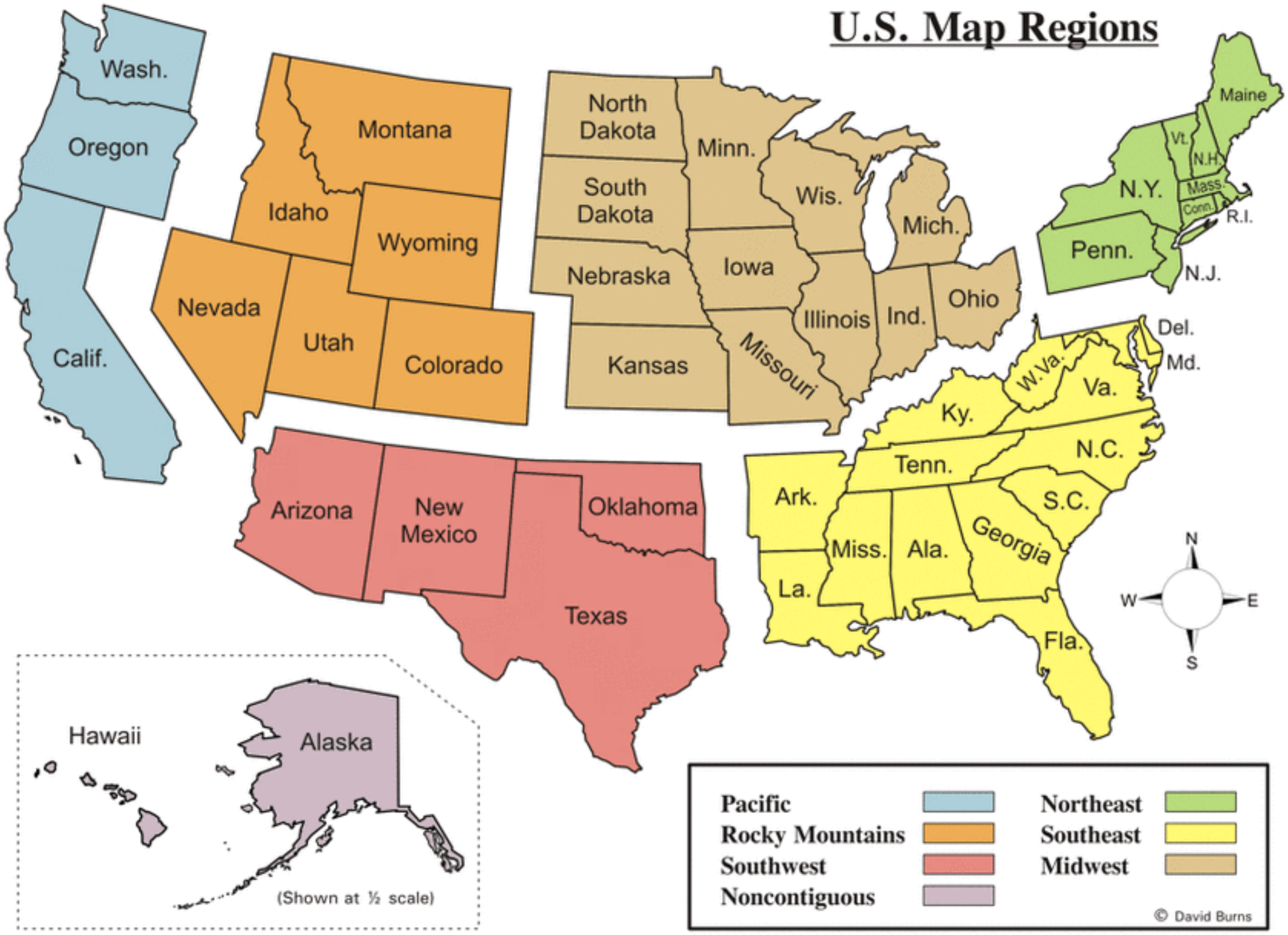

- ◯ Pacific
- ◯ Rocky Mountains
- ◯ Southwest

- Northeast
- Southeast
- Midwest
- Noncontiguous

Do you have prescribing capabilities?

- Yes
- No

Are you currently practicing or seeing patients/clients?

- Yes
- No

Do you conduct research in any capacity?

- Yes
- No

Have you ever taken a hallucinogen?

- Yes
- No
- Prefer not to answer

Cannabis (Marijuana)

These next questions focus on cannabis (marijuana).

Cannabis (Marijuana)

Please rate how strongly you agree with the following statements:

	Strongly disagree	Disagree	Neutral	Agree	Strongly agree
I have good knowledge of the potential therapeutic uses of cannabis.	O	O	O	O	O
I have good knowledge of the risks and side effects of cannabis.	O	O	O	O	O
I have good knowledge of the pharmacology of cannabis.	O	O	O	O	O
I have patients that use cannabis currently.	O	O	O	O	O
I would be open to using cannabis as a treatment for my patients.	O	O	O	O	O
	Strongly disagree	Disagree	Neutral	Agree	Strongly agree
I would like to receive training on using cannabis as a treatment with my patients.	O	O	O	O	O
Cannabis can be delivered safely in a clinical setting.	O	O	O	O	O
Cannabis shows promise in treating medical conditions.	O	O	O	O	O
Cannabis deserves further research for treatment of medical conditions.	O	O	O	O	O

	Strongly disagree	Disagree	Neutral	Agree	Strongly agree
Cannabis should be legally accessible for spiritual/religious use.	O	O	O	O	O

	Strongly disagree	Disagree	Neutral	Agree	Strongly agree
Cannabis should be legally accessible for recreational/non-medical use.	O	O	O	O	O
Cannabis should be legally accessible for supervised medical use.	O	O	O	O	O
Personal experience with cannabis is important to be able to use it clinically.	O	O	O	O	O

This is a survey about:

- O Hot dogs
- O Sports cars
- O Hallucinogens
- O Classic literature

Where has most of your knowledge on cannabis come from? (select all that apply)

- ☐ Formal clinical training
- ☐ Colleagues
- ☐ Academic literature
- ☐ Conference presentations or workshops
- ☐ Media (e.g., books, films, podcasts, social media, online forums)
- ☐ Informal conversations
- ☐ Past experience with patients
- ☐ Personal experience
- ☐ [ ] Other

What types of sources would you trust for training and information on therapeutic use of cannabis? (select all that apply)

- ☐ Professional organizations
- ☐ Pharmaceutical / Medical cannabis companies
- ☐ Academic research centers
- ☐ Experienced clinicians / practitioners
- ☐ Private training institutions
- ☐ ________ Other

In what settings do you think it would be appropriate to administer cannabis clinically? (choose all that apply)

- ☐ Private practice
- ☐ Hospital (outpatient)
- ☐ Hospital (inpatient)
- ☐ Detox / Drug rehabilitation facilities
- ☐ Specialized clinics
- ☐ Emergency Department
- ☐ At patient's home (unsupervised)
- ☐ At patient's home (supervised)
- ☐ None of these
- ☐ ________ Other

Please rate the importance of the following potential concerns of administering cannabis clinically to patients:

	Not at all concerned	Somewhat concerned	Moderately concerned	Very concerned	Extremely concerned
Psychosis	○	○	○	○	○
Addiction	○	○	○	○	○
Stigma	○	○	○	○	○
Financial cost / insurance coverage	○	○	○	○	○
Recreational use / misuse	○	○	○	○	○

	Not at all concerned	Somewhat concerned	Moderately concerned	Very concerned	Extremely concerned
	Not at all concerned	Somewhat concerned	Moderately concerned	Very concerned	Extremely concerned
Lack of trained providers	○	○	○	○	○
Time required to administer	○	○	○	○	○
Administration to patients with contraindications	○	○	○	○	○
Exploitation of patients	○	○	○	○	○
Other ______	○	○	○	○	○

Have you ever seen someone under the influence of cannabis? (select all that apply)

- ☐ No, never
- ☐ Yes, while they were seeking medical care
- ☐ Yes, in a recreational context
- ☐ Yes, in a research / clinical setting

How would you describe the experience(s) you observed?

- ○ Primarily positive
- ○ Somewhat positive
- ○ Neutral
- ○ Somewhat negative
- ○ Primarily negative

Knowledge Check

Current data indicate cannabis and cannabinoids (e.g. THC, CBD) may be useful for treating (select all that apply):

- ☐ Schizophrenia spectrum and other psychotic disorders
- ☐ Bipolar and related disorders
- ☐ Muscle spasms
- ☐ Pain symptoms and related disorders

- ☐ Substance-related and addictive disorders
- ☐ Nausea and vomiting
- ☐ Management of acute agitation

Risks of cannabis may include (select all that apply):

- ☐ Increased blood pressure and heart rate
- ☐ Dizziness
- ☐ Rash
- ☐ Nausea
- ☐ Nosebleed
- ☐ Constipation
- ☐ Psychosis

The primary mechanism by which cannabis is thought to work is (select all that apply):

- ☐ Activity at serotonin receptors
- ☐ Activity at dopamine receptors
- ☐ Activity at CB1 and CB2 receptors
- ☐ Activity at the norepinephrine receptors
- ☐ Activity at opioid receptors

Would you like to elaborate or provide any additional thoughts on the therapeutic use of cannabis?

- ○ Yes [ ]
- ○ No

Information & Resources on Cannabis

Currently, 49 states in the United States (US) and Washington, D.C. have approved some form of cannabinoid use, ranging from full recreational access for adults, to medical use [illegible]

of particular cannabinoid formulations for specific conditions (DISA, 2020). However, aside from the Food and Drug Administration (FDA) approved medications Marinol (for chemotherapy or AIDS-related nausea / weight-loss) and Epidiolex (for seizure reduction), rigorous data regarding clinical efficacy of medical cannabinoid products are lacking

(Levinsohn & Hill, 2020). Nevertheless, cannabinoids, which are known to interact with CB1 and CB2 receptors (Bow & Rimoldi, 2016), are increasingly being used across a wide range of conditions, including for pain (Stockings et al., 2018), sleep issues (Bachhuber et al., 2019), seizure disorders (Hussain et al., 2015), mental health conditions (e.g., depression, anxiety, post-traumatic stress disorder [PTSD] (Black et al., 2019), and cancer (Schleider et al., 2018), to name a few. To date, results surrounding efficacy are mixed, and much of the current observational and clinical research literature is limited by potential bias and insufficient data (Whiting et al., 2015). Potential risks may include tachycardia, euphoria, impaired judgment / concentration, psychosis, and dependence (Levinsohn & Hill, 2020).

References

Bachhuber, M., Arnsten, J. H., & Wurm, G. (2019). Use of cannabis to relieve pain and promote sleep by customers at an adult use dispensary. Journal of psychoactive drugs, 51(5), 400-404. https://www.tandfonline.com/doi/full/10.1080/02791072.2019.1626953

Black, N., Stockings, E., Campbell, G., Tran, L. T., Zagic, D., Hall, W. D., ... & Degenhardt, L. (2019). Cannabinoids for the treatment of mental disorders and symptoms of mental disorders: a systematic review and meta-analysis. The Lancet Psychiatry, 6(12), 995-1010. https://www.sciencedirect.com/science/article/abs/pii/S2215036619304018

Bow, E. W., & Rimoldi, J. M. (2016). The structure–function relationships of classical cannabinoids: CB1/CB2 modulation. Perspectives in medicinal chemistry, 8, PMC-S32171. https://journals.sagepub.com/doi/full/10.4137/PMC.S32171

DISA Global Solutions (2021). Map of marijuana legality by state. DISA Website. https://disa.com/map-of-marijuana-legality-by-state

Hussain, S. A., Zhou, R., Jacobson, C., Weng, J., Cheng, E., Lay, J., ... & Sankar, R. (2015). Perceived efficacy of cannabidiol-enriched cannabis extracts for treatment of pediatric epilepsy: a potential role for infantile spasms and Lennox–Gastaut syndrome. Epilepsy & Behavior, 47, 138-141.

https://www.sciencedirect.com/science/article/pii/S1525505015001572

Levinsohn, E. A., & Hill, K. P. (2020). Clinical uses of cannabis and cannabinoids in the United States. Journal of the neurological sciences, 411, 116717.

https://www.sciencedirect.com/science/article/pii/S0022510X20300538

Schleider, L. B. L., Mechoulam, R., Lederman, V., Hilou, M., Lencovsky, O., Betzalel, O., ... & Novack, V. (2018). Prospective analysis of safety and efficacy of medical cannabis in large unselected population of patients with cancer. European journal of internal medicine, 49, 37-43.
https://www.sciencedirect.com/science/article/pii/S0953620518300232

Stockings, E., Campbell, G., Hall, W. D., Nielsen, S., Zagic, D., Rahman, R., ... & Degenhardt, L. (2018). Cannabis and cannabinoids for the treatment of people with chronic noncancer pain conditions: a systematic review and meta-analysis of controlled and observational studies. Pain, 159(10), 1932-1954.
https://journals.lww.com/pain/fulltext/2018/10000/Cannabis_and_cannabinoids_for_the_tre

Whiting, P. F., Wolff, R. F., Deshpande, S., Di Nisio, M., Duffy, S., Hernandez, A. V., ... & Kleijnen, J. (2015). Cannabinoids for medical use: a systematic review and meta-analysis. Jama, 313(24), 2456-2473. https://jamanetwork.com/journals/jama/article-abstract/2338251

Quality Check

Did you have any issues completing the survey that would make your responses inaccurate or invalid?

- ○ [ ] Yes (please explain)
- ○ No

Did you have any trouble understanding the questions that would make your responses inaccurate or invalid?

- ○ [ ] Yes (please explain)
- ○ No

Did you answer the questions honestly and accurately to the best of your knowledge?

- Yes
- No

For Individuals who indicate their responses are inaccurate or invalid:

We sincerely appreciate your interest in this survey and your willingness to volunteer your time. Unfortunately, you do not currently meet all of the necessary criteria to participate.

Because you indicated issues with your survey responses, these will not be included in the final analysis.

With gratitude for your interest,
The Johns Hopkins Center for Psychedelic and Consciousness Research

JOHNS HOPKINS
Center for Psychedelic & Consciousness Research

For Individuals who complete the survey and indicate their responses are valid:

You have completed the survey! Thanks for your response!

We sincerely appreciate your time and effort completing the survey and look forward to publishing the results.

With gratitude,
The Johns Hopkins Center for Psychedelic and Consciousness Research

JOHNS HOPKINS
Center for Psychedelic & Consciousness Research

Powered by Qualtrics
